# Direct RNA sequencing-based multi-omics profiling reveals transcriptional and post-transcriptional remodeling in mice with diet-induced atherosclerosis

**DOI:** 10.3389/fcell.2026.1953461

**Published:** 2026-10-08

**Authors:** Xin Liu, Xueyu Han, Wenjun Qiu, Fengming Guo, Yan Guo, Yipeng Gao, Yuxin Liu, Ruichen Xu, Ke Hu, Bo Shen

**Affiliations:** 1 Department of Cardiology, Renmin Hospital of Wuhan University, Wuhan, China; 2 Hubei Key Laboratory of Metabolic and Chronic Diseases, Wuhan, China; 3 Department of Vascular Surgery, Renmin Hospital of Wuhan University, Wuhan, China; 4 State Key Laboratory of Neurology and Oncology Drug Development, Nanjing, China

**Keywords:** 3′-end processing, alternative splicing, atherosclerosis, direct RNA sequencing, multi-omics, RNA modification

## Abstract

**Introduction:**

Atherosclerosis is a vascular disease whose pathogenesis involves complex transcriptional and post-transcriptional regulation. Conventional bulk RNA-seq captures gene-level expression but does not fully resolve isoform diversity and RNA regulatory features.

**Methods:**

Male apolipoprotein E-deficient mice were fed normal chow (NC) or a Western diet (AS) for 16 weeks to establish diet-induced atherosclerosis. Aortic tissues were analyzed by direct RNA sequencing (DRS), bulk RNA-seq, and Assay for Transposase-Accessible Chromatin using sequencing (ATAC-seq). DRS data were used to reconstruct and quantify isoforms and to assess differential transcript expression, RNA modification, alternative splicing, alternative polyadenylation, and poly(A) tail length.

**Results:**

DRS generated a full-length aortic transcriptome comprising 35,587 transcripts, including 18,081 novel transcripts and 1,727 differentially expressed transcripts (DETs). Genes corresponding to known DETs were predominantly enriched in immune and inflammatory processes, whereas genes corresponding to novel DETs were primarily associated with lipid metabolism. Integration of ATAC-seq and DRS identified 13 DETs linked to altered chromatin accessibility. DRS further revealed extensive remodeling of RNA modifications, alternative splicing, and 3′-end processing, including dynamic changes in N6-methyladenosine, 5-methylcytosine, and adenosine-to-inosine. Additionally, 459 differential alternative splicing events and 1,320 differential polyadenylation sites were identified. A total of 613 genes exhibited altered 3′untranslated region length with coordinated shifts in poly(A) tail length.

**Discussion:**

By integrating DRS with bulk RNA-seq and ATAC-seq, this study establishes a multi-layered landscape of RNA transcriptional and post-transcriptional regulation in atherosclerosis. These findings provide a resource for future mechanistic investigations and for the development of RNA-targeted strategies in vascular disease.

## Graphical Abstract

**Figure:**
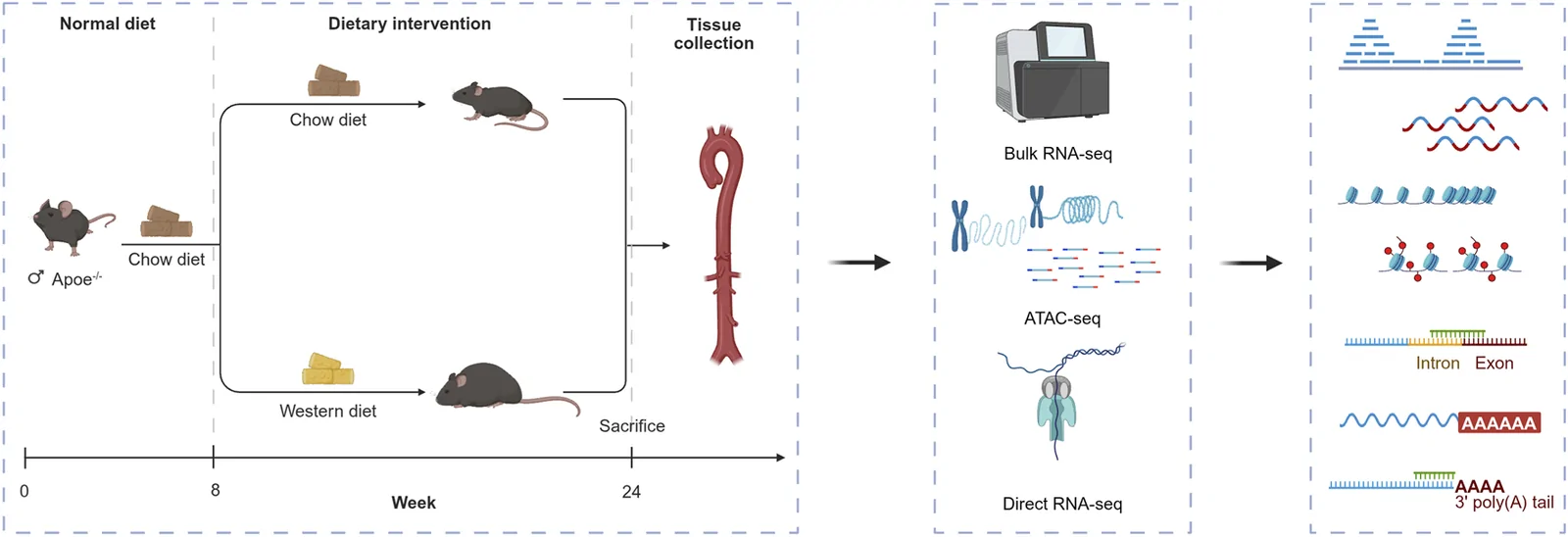

## 1 Introduction

Atherosclerosis is a chronic inflammatory disease of the vascular wall and constitutes the shared pathological foundation of multiple cardiovascular diseases. Its progression involves a sequence of pathological processes, including endothelial dysfunction, inflammatory cell recruitment and lipid deposition, plaque formation and maturation, and plaque instability that predisposes to rupture, ultimately leading to thrombosis and adverse cardiovascular events (Libby, 2021). Epidemiological evidence indicates an increasing burden of atherosclerosis-related cardiovascular diseases, particularly among older adults (Liberale et al., 2025). Given the complexity of atherosclerosis pathogenesis, a comprehensive perspective on gene expression regulation is essential for elucidating disease progression.

Most previous studies have relied on short-read RNA-seq, which enables accurate gene-level expression quantification (Xu et al., 2024). However, the limited read length complicates the precise reconstruction of complex genomic loci and the analysis of full-length transcripts, thereby restricting systematic characterization of RNA diversity and transcript structural complexity. Accumulating evidence indicates that transcript isoform switching and post-transcriptional regulation play critical roles in atherosclerosis (Stopa et al., 2025). Post-transcriptional regulatory mechanisms, including RNA modification, alternative splicing, and alternative polyadenylation (APA), can generate transcript isoforms with distinct coding sequences or untranslated regions, thereby influencing protein output, RNA stability, subcellular localization, and translation efficiency (Corbett, 2018). Conventional short-read RNA-seq offers insufficient resolution for these post-transcriptional regulatory events, limiting a more comprehensive understanding of the RNA regulatory landscape in atherosclerosis.

Direct RNA sequencing (DRS) on the Nanopore platform enables the direct reading of native RNA molecules without reverse transcription or amplification, thereby allowing comprehensive full-length isoform characterization while preserving information on RNA modifications (Kim et al., 2025; Sun et al., 2025). Additionally, Assay for Transposase-Accessible Chromatin using sequencing (ATAC-seq) provides genome-wide mapping of chromatin accessibility and identifies putative regulatory regions. The integration of transcriptome profiling with ATAC-seq facilitates the association of gene expression alterations with chromatin accessibility remodeling, thereby offering additional insight into transcriptional regulatory mechanisms (Ranzoni et al., 2021).

Building on this background, DRS, bulk RNA-seq, and ATAC-seq were applied to systematically profile an apolipoprotein E-deficient (*Apoe* ^*−/−*^) mouse model of atherosclerosis. By generating a full-length transcriptome atlas of the aorta, differential expression was quantified at both gene and transcript levels. Chromatin accessibility alterations, RNA modification, and transcript-level variation were further integrated to identify putative regulatory sites and transcripts associated with atherosclerosis, thereby offering a more comprehensive understanding of the disease.

## 2 Materials and methods

### 2.1 Animals

Male *Apoe* ^*−/−*^ mice were obtained from Guangdong Kaishi Biotechnology Co., Ltd. After 1 week of adaptive feeding, 8-week-old *Apoe* ^*−/−*^ mice in good general health were randomly assigned to two groups: the control group (NC, n = 9) and the atherosclerosis group (AS, n = 9). The diet-induced atherosclerosis model was established following the American Heart Association scientific statement on the design and reporting of atherosclerosis studies (Daugherty et al., 2017). The NC group was fed sterile standard chow, whereas the AS group received a sterile Western diet. The diet-induced atherosclerosis model was established over 16 weeks. Mice were monitored daily for general health and signs of distress. At the experimental endpoint, aortic tissues were harvested for subsequent analyses. Briefly, following dissection and rinsing in ice-cold phosphate-buffered saline, aortas from three mice within the same group were pooled to generate one biological replicate. Three pooled biological replicates were prepared for each group. Each pooled biological replicate was subsequently divided into three portions for DRS, bulk RNA-seq, and ATAC-seq, respectively, thereby enabling cross-platform analyses from the same biological source. All animals were housed under specific pathogen-free conditions with a 12-h light-dark cycle (20 °C–25 °C; 50% ± 5% humidity) in individually ventilated cages with unrestricted access to water. All experimental procedures were approved by the Ethics Committee of Wuhan University Renmin Hospital (Approval No. WDRM 20260001) and followed the Guidelines for the Care and Use of Laboratory Animals (NIH Publication No. 82-23, revised 1996).

### 2.2 RNA extraction and bulk RNA-seq library preparation

Total RNA was extracted from aortic tissues using TRIzol reagent (Thermo Fisher Scientific, cat. #15596026CN, United States), and subsequently treated with DNase I (New England Biolabs, cat. #M0303L, United States) according to the manufacturer’s instructions. RNA purity and integrity were evaluated using a Nanodrop™ OneC spectrophotometer (Thermo Fisher Scientific, United States) and the LabChip GX Touch system (Revvity, United States). The Qubit 3.0 Fluorometer (Life Technologies, United States) and QubitTM RNA Broad Range Assay kit (Thermo Fisher Scientific, cat. #Q10210, United States) were used to determine the RNA concentration and quality. The KC™ mRNA Library Prep Kit (Seqhealth, China) was used to construct the RNA sequencing library following the manufacturer’s protocol, with total RNA as input material. Library preparation included enrichment of PCR-amplified fragments ranging from 200 to 500 base pairs (bp) in length. After quantification, the enriched libraries were sequenced in PE150 paired-end mode on a DNBSEQ-T7 platform (MGI, China).

### 2.3 Bulk RNA-seq analysis

Raw sequencing reads containing more than two ambiguous bases (N) were removed. Adapter sequences and low-quality bases were trimmed from the raw reads using Cutadapt (v3.4) (Martin, 2011) and FASTX-Toolkit (v0.0.14). Reads shorter than 20 nt were also excluded. The resulting clean reads were aligned to the mouse reference genome (GRCm39) using HISAT2 (v2.2.1) (Kim et al., 2015), allowing up to four mismatches. Only uniquely mapped reads were retained for downstream analyses. Gene-level raw counts generated by Salmon (v1.7.0) were used as input for differential expression analysis, whereas transcripts per million (TPM) values were used for visualization. Differential expression analysis was conducted using DESeq2 (v1.30.1), with false discovery rate (FDR, Benjamini–Hochberg–adjusted P-value) < 0.05 and |log_2_fold change (FC)| ≥ 1 used as the cutoff criteria for identifying differentially expressed genes (DEGs).

### 2.4 ATAC-seq library preparation and analysis

Fresh aortas were transferred to a pre-cooled tissue grinder containing lysis buffer and homogenized. Subsequently, nuclei were isolated by density-gradient centrifugation to obtain purified nuclei. Tn5 transposition was performed using the Hyperactive ATAC-seq Library Prep Kit (Vazyme, cat. #TD711, China). Purified nuclei were incubated with Tn5 transposase at 37 °C for 30 min. Tagmented DNA was recovered by DNA Extraction Beads and amplified by PCR. The library products were enriched, quantified, and sequenced on the DNBSEQ-T7 platform (MGI, China) in PE150 paired-end mode. Paired-end reads were aligned to the GRCm39 genome using Bowtie2 (v2.4.4). Duplicate reads were removed using Sambamba (v0.8.1). Only reads mapped to regular chromosomes were retained. SAM files were converted to BAM format, sorted, and indexed by SAMtools (v1.7). Peak calling was performed using MACS2 (v2.2.7.1). The processed BAM files were subsequently used to extract read counts within peaks. Hypergeometric Optimization of Motif EnRichment (HOMER) software (v5.1) (Heinz et al., 2010) was employed to identify enriched binding motifs within peaks. DESeq2 was used to identify differentially accessible peaks. A P-value < 0.05 was applied as the cutoff criterion for identifying different peaks.

### 2.5 Nanopore DRS library preparation and sequencing

Total RNA was used to enrich polyadenylated RNA following the manufacturer’s instructions for the KAPA mRNA HyperPrep Kit (Roche, cat. #08098115702, Switzerland). For DRS library preparation, enriched poly(A) + RNA was subjected to adapter ligation and reverse transcription. Briefly, 9 μL poly(A)+ RNA was combined with 3 μL NEBNext® Quick Ligation Reaction Buffer (New England Biolabs, cat. #B6058, United States), 1 μL RT Adapter (Oxford Nanopore Technologies, cat. #SQK-RNA004, United Kingdom), and 2 μL T4 DNA Ligase (New England Biolabs, cat. #M0202, United States), followed by incubation at 25 °C for 10 min to ligate RNA adapters. For reverse transcription, 8 μL of 5 × first-strand buffer (New England Biolabs, cat. #M0681, United States), 2 μL of 10 mM Deoxynucleotide (dNTP) Solution Mix (New England Biolabs, cat. #N0447, United States), 9 μL of nuclease-free water, 4 μL of 0.1 M dithiothreitol, and 2 μL Induro® Reverse Transcriptase (New England Biolabs, cat. #M0681, United States) were added, followed by incubation at 50 °C for 50 min and at 70 °C for 10 min. The reverse-transcribed RNA was purified using 1.8 × Agencourt RNAClean XP beads (Beckman, cat. #A63987, United States). Sequencing adapters were ligated using 8 μL NEBNext® Quick Ligation Reaction Buffer (New England Biolabs, cat. #B6058, United States), 6 μL RNA Ligation Adapter (Oxford Nanopore Technologies, cat. #SQK-RNA004, United Kingdom), and 3 μL T4 DNA Ligase (New England Biolabs, cat. #M0202, United States), followed by bead purification and washing. The final reaction mixture was combined with 50 µL RNA Running Buffer (Oxford Nanopore Technologies, cat. #SQK-RNA004, United Kingdom) and 35 µL nuclease-free water, and then loaded onto a PromethION RNA flow cell (Oxford Nanopore Technologies, United Kingdom) for 48–72 h of sequencing on the PromethION sequencer (Oxford Nanopore Technologies, United Kingdom).

### 2.6 Nanopore DRS analysis

The raw data were generated in POD5 format, which contains raw sequencing signals. Dorado (v1.0.2) was used for base-calling and modify-calling and to convert POD5 data into BAM format, which includes base information, sequencing quality metrics, modified-base probability tags and estimated poly(A) length information. Quality filtering was performed using SeqKit (v2.3.1) (Shen et al., 2024) to retain only reads with a quality score ≥ 10 and length ≥ 50 bp for subsequent analyses. Filtered reads were aligned to the Ensembl reference genome (GRCm39) using Minimap2 (v2.27-r1193) in splice-aware mode. The SAMtools fastq tool (v1.7) was used to extract high-quality sequences from BAM files into FASTQ format.

The FLAIR pipeline (v2.2.0) (Tang et al., 2020) was applied to identify full-length isoforms which were filtered according to the following criteria: (1) transcripts with a 5′end located within 100 bp of transcription start site (TSS) annotated by refTSS and/or TSSclassifier (“relaxed” or “strict”) based on the FANTOM CAGE peak (Abugessaisa et al., 2019); (2) transcripts supported by at least three full-length reads, with each supporting read covering at least 80% of the transcript length and spanning at least 25 bp of both the first and last exons. These criteria were applied to improve the confidence of the reconstructed transcript models. The SQANTI3 pipeline (v5.4) (Tardaguila et al., 2018) was subsequently used with default parameters to remove transcripts considered artifacts and to classify structural categories. Structural novelty was defined relative to the Ensembl/GRCm39 reference annotation. Transcripts that did not fully match reference transcript models were classified by SQANTI3 into novelty categories including novel-in-catalog, novel-not-in-catalog, genic, genic_intron, antisense, and intergenic transcripts. Transcripts containing the CAGE site were extracted from the annotated reference dataset of mouse TSS. Read density distributions in BAM files were analyzed using Deeptools (v3.5.6) (Ramírez et al., 2014), focusing on 3 kb upstream and downstream of gene TSS. Heatmaps of read density distribution were generated. To evaluate the accuracy of abundance estimation at transcript and gene resolution, the quasi-mapping mode of Salmon (v1.7.0) was used to estimate TPM for each transcript and gene in each sample. Differential expression analysis was performed using DESeq2 (v1.30.1), with |log_2_FC| ≥ 1 and FDR < 0.05 as the statistical cutoff criteria for identifying DEGs and differentially expressed transcripts (DETs).

### 2.7 RNA modification detection and analysis

Sequencing POD5 files were base-called using Dorado (v1.0.2) to obtain modification site information for N6-methyladenosine (m^6^A), 5-methylcytosine (m^5^C), and adenosine-to-inosine (A-to-I). RNA modification calls were subsequently processed using Modkit (v0.5.0) at both the transcript and genome levels. For transcript-level modification calling, the command “modkit extract calls--mapped-only--no-headers--edge-filter 20” was used. Modification calls located within 20 bp of either read end were excluded to reduce potential edge-associated calling artifacts. Only calls with a modification probability (call_prob) > 0.7 were retained, and transcript-level sites were further required to have a minimum coverage of > 10 reads. For genome-level modification calling, the command “modkit pileup sample. sorted.bam genome_call.sample.bed--ref GRCm39. genome.fa” was used. Genome-level sites were retained if the sequencing coverage was >10 reads and at least one modified-base call was detected at the corresponding position. The modification ratio at each retained site was then calculated for downstream analyses. Rtracklayer (v1.66.0) (Lawrence et al., 2009) was used to annotate modification sites according to transcript features, including the 5′untranslated region (UTR), coding sequence (CDS), and 3′UTR. Differential modification site analysis between the AS and NC groups was performed using MethylKit (v1.33.3) (Akalin et al., 2012). Individual sites with a modification ratio difference > 10% and a P-value < 0.05 were defined as hyper-sites or hypo-sites.

### 2.8 Alternative splicing analysis

Alternative splicing events (ASEs), including retained intron (RI), skipping exon (SE), alternative 3′splice site, alternative 5′splice site, mutually exclusive exon (MX), alternative first exon (AF), and alternative last exon (AL), were identified and classified using SUPPA2 (v2.3) (Trincado et al., 2018). Percent spliced-in (PSI) values for each ASE were calculated. ASEs with an absolute differential PSI (dPSI) > 0.05 and a P-value < 0.05 were considered statistically significant.

### 2.9 APA and poly(A) tail length analysis

APA analysis was conducted using TAPAS (v2018.5.26) (Arefeen et al., 2018) to identify polyadenylation sites (PASs). Differential APA between AS and NC groups was assessed, and events with |log_2_FC| ≥1 and P < 0.05 were considered statistically significant. Changes in 3′UTR length were inferred from shifts in PAS usage: increased distal PAS usage (negative log_2_FC) indicated 3′UTR lengthening, whereas increased proximal PAS usage (positive log_2_FC) indicated 3′UTR shortening. Sequences within 50 bp upstream of PASs were extracted and analyzed using STREME (v5.5.9) to identify enriched sequence motifs. Poly (A) tail length was estimated using Dorado (v1.0.2). Poly(A) tail length data from all reads were extracted and categorized according to annotated transcripts. Significant changes in poly (A) tail length between groups were defined using a |log_2_FC| ≥ 0.585 (corresponding to a ≥ 1.5-fold change) and a P-value < 0.05 as the cutoff criteria.

### 2.10 Pathway enrichment analysis

Gene Ontology (GO) term and Kyoto Encyclopedia of Genes and Genomes (KEGG) pathway enrichment analyses were performed using KOBAS 2.0 (v2.0) (Xie et al., 2011). For transcript-level analyses, transcripts were first mapped to their corresponding genes using Ensembl gene identifiers, and enrichment analyses were subsequently performed on the resulting gene sets. Multiple transcripts originating from the same gene were therefore represented by the same gene-level functional annotation. Transcripts that could not be assigned to an annotated gene were excluded from standard GO/KEGG enrichment analyses. Clustering analysis of enriched pathways was conducted using pheatmap (v1.0.12) in R (v4.3.3) based on Euclidean distance for visualization. Statistical significance was evaluated using the hypergeometric test. Terms with an FDR <0.05 were considered significantly enriched.

### 2.11 Statistical analysis

All statistical analyses and data visualizations were performed in R (v4.3.3) using RStudio. Data are presented as the mean ± standard error of the mean (SEM). Differential expression (DEGs/DETs) and differentially accessible regions (DARs) were analyzed using DESeq2 (v1.30.1). Differential splicing was quantified using SUPPA2 (v2.3). Differential RNA modification sites were tested using MethylKit (v1.33.3) and APA was analyzed using TAPAS (v2018.5.26). A P-value <0.05 was considered statistically significant.

## 3 Results

### 3.1 DRS establishes a full-length aortic transcriptome atlas in diet-induced atherosclerosis

Eight-week-old male *Apoe* ^*−/−*^ mice were fed a Western diet to induce atherosclerosis and the aortic transcriptome was characterized through an integrated analysis combining DRS, bulk RNA-seq, and ATAC-seq. Comparison of log_10_ (TPM + 1) distributions for genes and transcripts quantified by DRS and bulk RNA-seq revealed that bulk RNA-seq produced lower gene-level expression estimates than DRS, whereas transcript-level values derived from DRS were reduced relative to the corresponding gene-level measurements (Supplementary Figure S1). These discrepancies are likely attributable to differences in read length, library preparation, and quantification strategies between the two sequencing platforms, suggesting that DRS offers enhanced resolution for detecting low-abundance transcript isoforms in arterial tissue.

Across six samples, DRS detected comparable numbers of genes and transcripts, with slightly higher counts observed in NC than in AS (Figure 1A). The mean transcript length was greater in NC than in AS (Figure 1B), indicating transcript structural remodeling under atherosclerotic conditions. Based on the annotated mouse reference genome, of the 35,587 transcripts detected in this study, 17,506 overlapped with the reference annotation, whereas 18,081 transcripts were not represented in the reference set, demonstrating that DRS significantly expands existing transcript-level annotations (Figure 1C). Structural category annotation identified 19,893 known and 15,694 novel transcripts. Among these, full-splice match transcripts correspond to fully annotated isoforms, whereas incomplete-splice match transcripts represent partial splice matches of known genes. Novel-in-catalog, novel-not-in-catalog, genic, and genic_intron denote novel transcript isoforms of known genes; fusion denotes fusion transcripts; and antisense and intergenic transcripts are likely derived from previously unannotated genes (Figures 1D,E). At the gene level, 15,368 genes were identified, including 14,644 known genes and 724 novel genes (Figure 1E).

**FIGURE 1:**
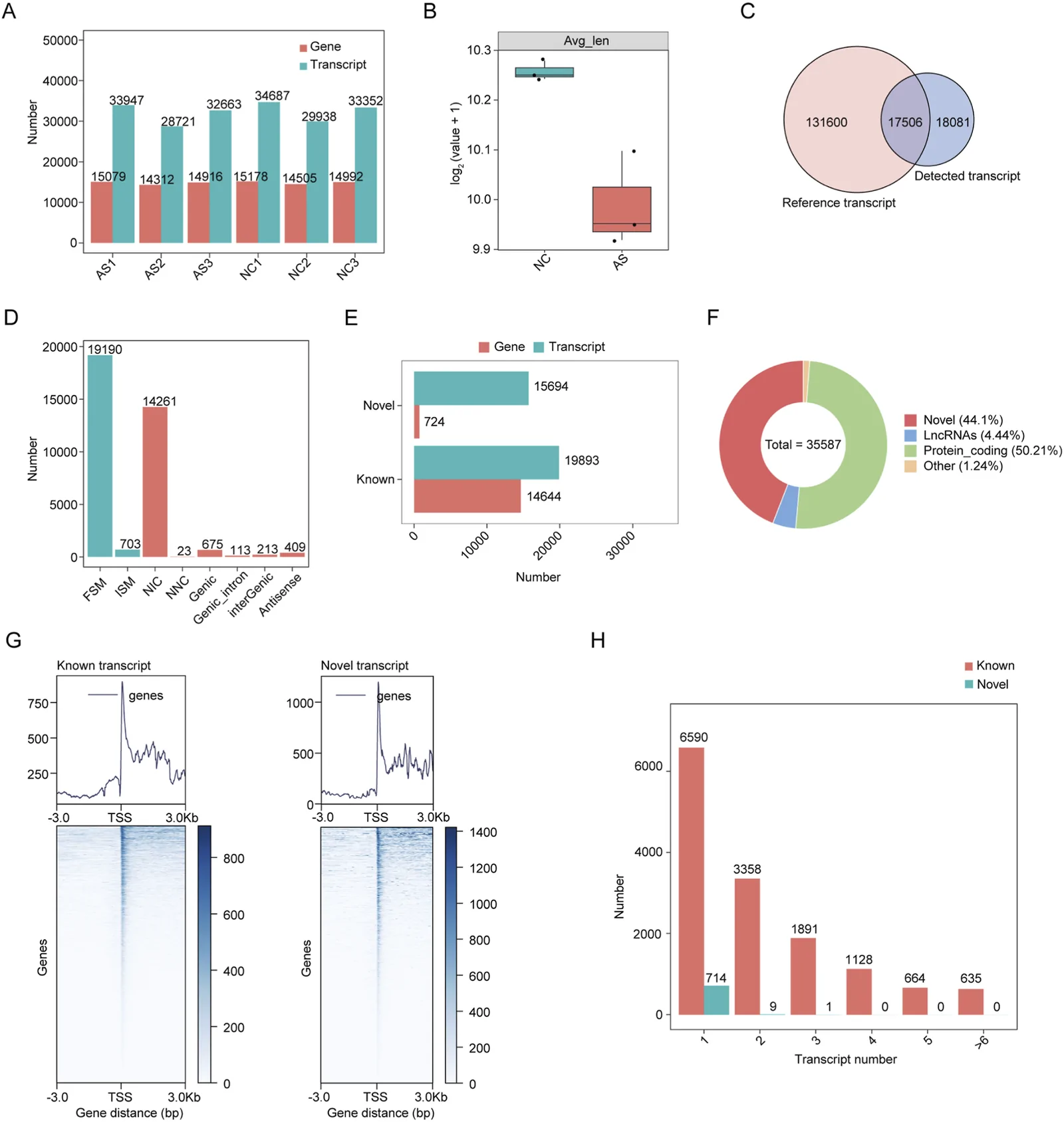
Nanopore direct RNA sequencing (DRS) establishes a full-length transcriptome landscape in atherosclerosis. **(A)** Number of genes and transcripts detected by DRS in each sample. **(B)** Distribution of average transcript length (log_2_ (value + 1)) in NC and AS samples (n = 3 biological replicates per group). **(C)** Venn diagram illustrating the overlap between DRS-identified transcripts and the Ensembl/GRCm39 reference transcript annotation. **(D)** Structural classification of DRS-identified transcripts (FSM, full-splice match; ISM, incomplete-splice match; NIC, novel-in-catalog; NNC, novel-not-in-catalog). **(E)** Numbers of known and novel genes and transcripts identified by DRS. **(F)** Proportion of transcript biotype composition (lncRNAs, long non-coding RNAs). **(G)** Read density surrounding transcription start site (TSS, ± 3 kb) for known and novel transcripts. **(H)** Number of transcripts per gene in known and novel sets.

Analysis of transcript composition by annotation type demonstrated that 50.21% were protein-coding, 4.44% were long non-coding RNAs, and 44.1% were novel, reflecting substantial isoform-level structural diversity in the aorta (Figure 1F). Read density profiles surrounding TSSs showed pronounced enrichment for both known and novel transcripts (Figure 1G), supporting the accuracy of the constructed transcript structures. Genes harboring multiple isoforms (> 4 transcripts) were predominantly annotated, while novel transcripts were more commonly observed in genes with fewer isoforms (Figure 1H). Long-read sequencing effectively provides complementary advantages over conventional approaches by enabling precise resolution of complex transcript structural features.

### 3.2 Gene-level and transcript-level differential analysis uncovers multi-layered expression changes in atherosclerosis

Transcript-level differential expression analysis of DRS data identified 1,727 significant DETs, including 1,333 annotated transcripts and 394 novel transcripts (Supplementary Figure S2A). GO analysis of genes corresponding to annotated DETs showed enrichment primarily in immune and inflammatory biological processes (Supplementary Figure S2B). In contrast, genes corresponding to novel DETs were enriched in lipid and fatty acid metabolic processes, the tricarboxylic acid cycle, and positive regulation of cold-induced thermogenesis. These findings indicate that, beyond classical immune-inflammatory responses, atherosclerotic states are also associated with metabolism-related transcriptional alterations that are more prominently captured among novel transcripts (Supplementary Figure S2C). At the gene level, DRS detected 1,258 DEGs, whereas bulk RNA-seq identified 2,693 DEGs (Supplementary Figure S2D). DRS and bulk RNA-seq shared 939 DEGs, representing 74.6% of DRS DEGs but only 34.9% of bulk DEGs, suggesting substantial concordance in gene-level differential expression while bulk RNA-seq detected additional DEGs unique to the bulk dataset.

To link transcript- and gene-level signals in DRS, we mapped DETs to their corresponding genes, identifying 1,114 genes overlapping between the DEG and DET sets (Figure 2A), along with 321 genes significant exclusively at the transcript level (DET-only) and 144 genes significant exclusively at the gene level (DEG-only). The DET-only category suggests that gene-level aggregation may obscure isoform-resolved regulation, as certain genes do not exhibit significant changes at the gene level yet display marked variation among individual transcript isoforms. Consistently, DET-only genes had more isoforms per gene than either the shared group or the DEG-only group (Figure 2B). Such isoform-specific alterations occur more frequently in isoform-rich genes and may be masked by gene-level aggregation, emphasizing the complementary contribution of transcript-level analysis.

**FIGURE 2:**
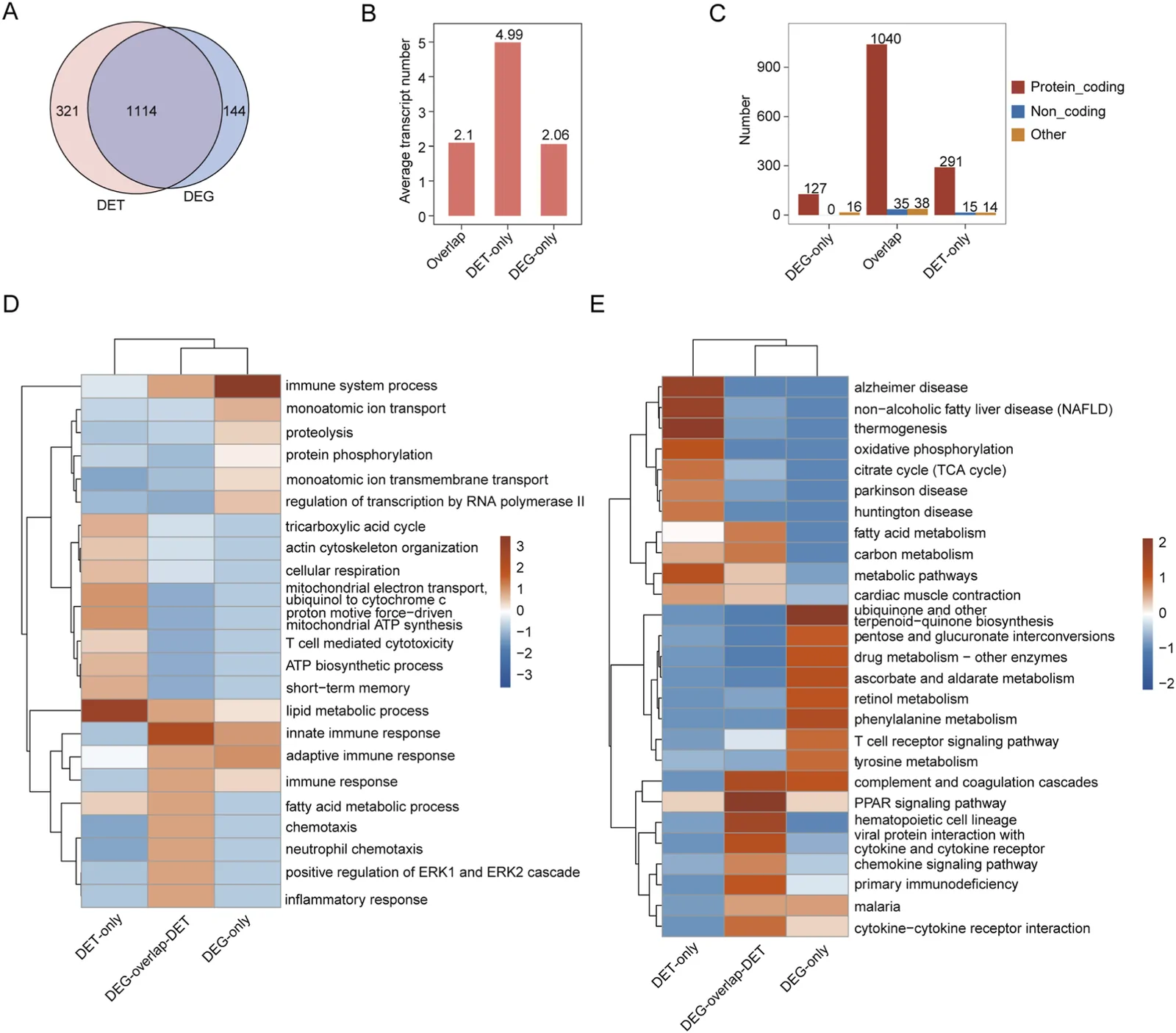
DRS-based differential expression analysis at the gene and transcript levels. **(A)** Venn diagram illustrating the overlap between genes corresponding to differentially expressed transcripts (DETs) and differentially expressed genes (DEGs). **(B)** Number of transcripts per gene within each overlapping category. **(C)** Biotype composition within each overlapping category. **(D)** Hierarchical clustering heatmap of Gene Ontology (GO) biological process enrichment for each overlapping category. **(E)** Hierarchical clustering heatmap of Kyoto Encyclopedia of Genes and Genomes (KEGG) pathway enrichment for each overlapping category.

Gene biotype distributions were comparable across the DET-only, DEG-only, and shared sets, with protein-coding genes accounting for the majority (Figure 2C). GO enrichment analysis demonstrated partial overlap in biological processes among these gene groups, while also revealing distinct functional signatures. DET-only genes were enriched in metabolic and energy-related processes, whereas DEG-only genes were enriched in immune-related processes (Figure 2D). KEGG pathway analysis similarly associated DEG-only genes with immune pathways, including complement and coagulation cascades (Figure 2E). Collectively, these findings indicate that transcript-level analysis provides information beyond gene-level differential expression thereby enabling a more comprehensive characterization of atherosclerosis-related transcriptional alterations.

### 3.3 ATAC-seq and DRS integration links chromatin accessibility changes with transcript expression dynamics

ATAC-seq identified 351 DARs, including 88 regions with increased accessibility (up-DARs) and 263 regions with decreased accessibility (down-DARs). These DARs were annotated to 86 and 236 genes, respectively (Supplementary Figure S3A). Up-DARs were preferentially localized to promoter regions, whereas down-DARs were predominantly enriched within intronic and intergenic regions (Supplementary Figure S3B), indicating chromatin accessibility remodeling associated with diet-induced atherosclerosis at promoters and putative distal regulatory regions. To examine whether DRS-defined transcript alterations correlate with chromatin accessibility, DETs were intersected with DARs, yielding 13 DETs accompanied by nearby accessibility changes (Figure 3A). These transcripts corresponded to genes involved in vascular structure and metabolism, including *Acta2*, *Tagln*, *Zyx*, *Lmod1*, *Gramd1b*, and *Scd1*. Notably, changes in promoter accessibility exhibited directional consistency with the corresponding transcript expression differences between NC and AS groups. Bulk RNA-seq coverage further confirmed robust transcriptional signals in these regions, supporting an association between transcript-level variation and adjacent chromatin accessibility.

**FIGURE 3:**
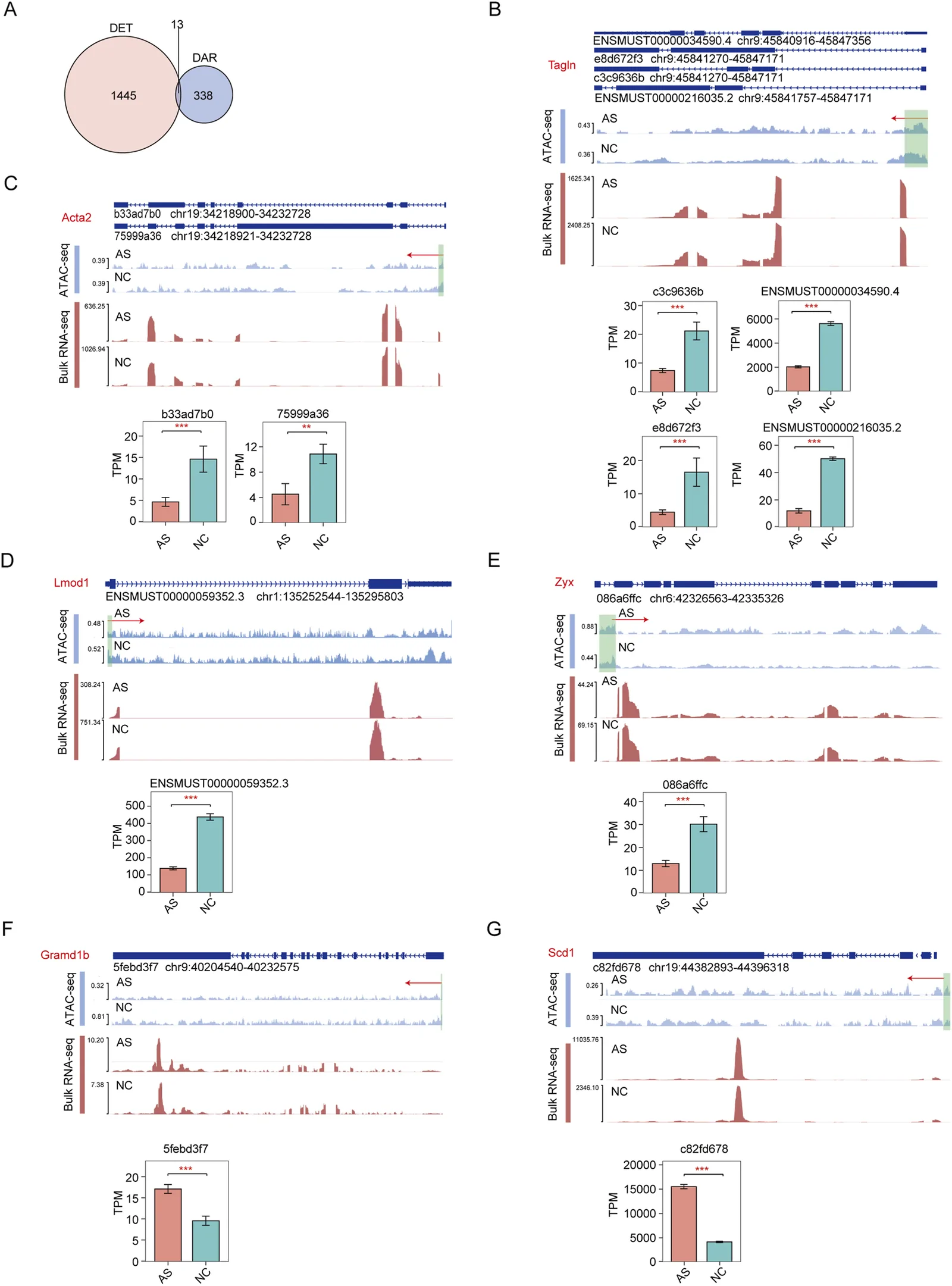
Integration of differential chromatin accessibility and transcript expression. **(A)** Venn diagram illustrating the overlap between DETs and differentially accessible regions (DARs). **(B–G)** Representative loci with genome browser tracks and quantitative summaries. Top: Schematic representation of transcripts identified by DRS. Middle: Assay for Transposase-Accessible Chromatin using sequencing (ATAC-seq) signal tracks (blue) and bulk RNA-seq coverage tracks (red) for the AS and NC groups. Quantitative signal scales are shown for each track, and differentially accessible regions are highlighted in the ATAC-seq tracks. Bottom: Relative transcript expression levels derived from DRS. Panels include *Tagln* **(B)**, *Acta2* **(C)**, *Lmod1* **(D)**, *Zyx* **(E)**, *Gramd1b* **(F)**, and *Scd1* **(G)**. Transcript identifiers with non-standard alphanumeric codes (e.g., e8d672f3) represent novel transcript models reconstructed by the FLAIR/SQANTI3 pipeline that are not present in the Ensembl reference annotation (GRCm39). Previously annotated transcripts are represented using standard Ensembl transcript identifiers (ENSMUST). Data are shown as mean ± standard error of the mean (SEM, n = 3 biological replicates per group). ***, *P* < 0.001; **, *P* < 0.01; *, *P* < 0.05.

Contractile vascular smooth muscle cell markers *Acta2*, *Tagln*, and *Lmod1* showed reduced transcript abundance together with decreased chromatin accessibility at their corresponding genomic regions in atherosclerotic tissue (Figures 3B–D) (Grootaert and Bennett, 2021; Nanda and Miano, 2012). Furthermore, *Zyx* has been implicated in regulating inflammation-related gene expression in endothelial cells and contributing to endothelial dysfunction. In this study, coordinated changes in its transcript levels and local accessibility signals were consistent with the inflammatory microenvironment characteristic of atherosclerosis (Figure 3E) (Wójtowicz et al., 2010). At metabolism-related loci, *Gramd1b* participates in cholesterol transport and absorption regulation, whereas *Scd1* regulates fatty acid metabolism and inflammatory responses (Figures 3F,G) (Xue et al., 2022; Luo et al., 2025; MacDonald et al., 2009). In atherosclerotic tissues, transcripts originating from these loci showed upregulated expression together with enhanced chromatin accessibility in adjacent regulatory regions, suggesting that transcriptional regulatory changes related to lipid handling and energy metabolism are also reflected in this model (Figures 3F,G).

### 3.4 Distribution characteristics and differential modification analysis of RNA modifications in atherosclerosis

As an important component of post-transcriptional regulation, RNA modifications contribute to the control of RNA fate (Roundtree et al., 2017). m^6^A, m^5^C, and A-to-I RNA modifications were profiled in atherosclerosis using DRS. All three modifications were consistently detected across six samples but differed in site abundance, modification ratios, and positional distribution along transcripts. Among these, m^6^A was the predominant modification, exhibiting the highest number of modified sites in all samples, with slightly fewer sites observed in AS2, NC1, and NC2 (Figure 4A). More than half of m^6^A sites displayed modification ratios above 0.5, and most transcripts contained more than 10 m^6^A-modified sites (Figure 4B). Transcript feature analysis indicated that m^6^A occurred mainly within the CDS region, particularly near the stop codon (Figure 4C). Compared with the AS group, m^6^A modification in the NC group exhibited a relative bias toward the 5′UTR (Supplementary Figure S4A). Differential analysis identified 266 m^6^A-enhanced sites and 619 m^6^A-attenuated sites in AS, indicating extensive remodeling of the m^6^A landscape in atherosclerosis (Figure 4D).

**FIGURE 4:**
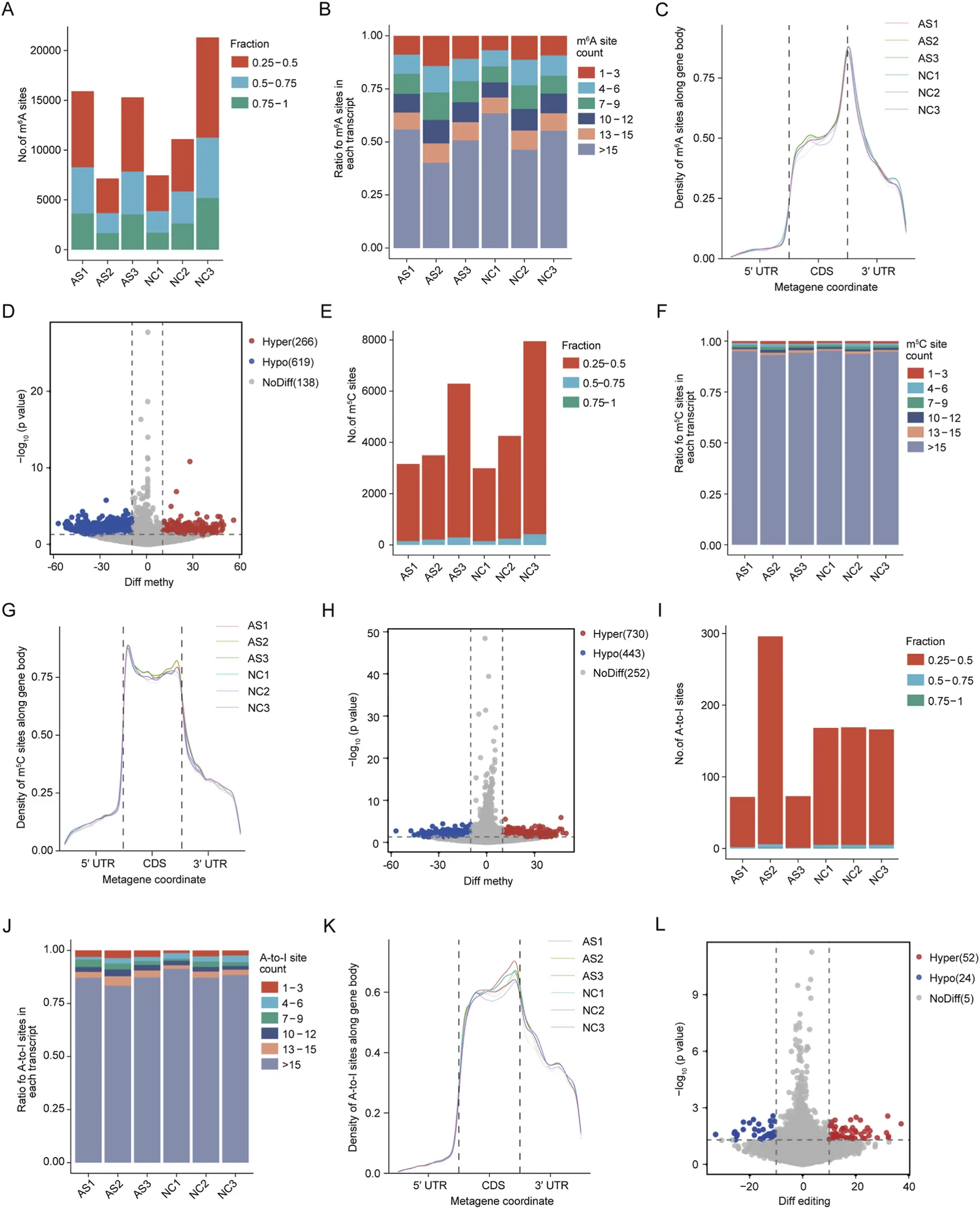
Analyzing transcriptome modifications via DRS in atherosclerosis. **(A)** Number of detected N6-methyladenosine (m^6^A)-modified sites in each sample. **(B)** Distribution of transcripts according to the number of m^6^A sites per transcript (binned) and modification fraction. **(C)** Metagene density of m^6^A sites across 5′untranslated region (UTR), coding sequence (CDS), and 3′UTR. **(D)** Differential analysis of m^6^A-modified sites between AS and NC, categorizing sites as hyper- or hypo-modified. **(E)** Number of detected 5-methylcytosine (m^5^C)-modified sites in each sample. **(F)** Distribution of transcripts by number of m^5^C sites per transcript (binned) and modification fraction. **(G)** Metagene density of m^5^C sites across 5′UTR, CDS, and 3′UTR. **(H)** Differential analysis of m^5^C-modified sites between AS and NC, categorizing sites as hyper- or hypo-modified. **(I)** Number of detected adenosine-to-inosine (A-to-I) editing sites in each sample. **(J)** Distribution of transcripts according to the number of A-to-I sites per transcript (binned) and editing fraction. **(K)** Metagene density of A-to-I sites across 5′UTR, CDS, and 3′UTR. **(L)** Differential analysis of A-to-I sites between AS and NC, categorizing sites as hyper- or hypo-edited.

In contrast to m^6^A, m^5^C modifications exhibit a more localized distribution across samples, with slightly fewer m^5^C-modified sites detected in the AS group than the NC group. Most m^5^C sites had modification ratios below 0.5, and the majority of transcripts contained over 15 m^5^C-modified sites (Figures 4E,F). Structural analysis showed that m^5^C was primarily enriched at both the start and end of the CDS region, forming a bimodal distribution pattern (Figure 4G; Supplementary Figure S4B). Differential analysis revealed 730 enhanced and 443 attenuated m^5^C sites in AS (Figure 4H). Although less prevalent than m^6^A, m^5^C experiences considerable regulatory reprogramming under the pathological conditions of atherosclerosis.

A-to-I RNA editing is infrequent in mammals but has received increasing attention for its regulatory functions in recent years (Zhang et al., 2024). Among the three modification types, A-to-I editing sites were the least abundant, with no sample exceeding 500 sites and slightly fewer sites detected in AS1 and AS3. Editing ratios were primarily concentrated in the 0.25–0.5 range, with most transcripts harboring more than 15 A-to-I-edited positions (Figures 4I,J). Its structural distribution resembles that of m^6^A, primarily enriched near the stop codon within the CDS region (Figure 4K; Supplementary Figure S4C). Compared with the NC group, the AS group exhibited enhanced editing at 52 sites and reduced editing at 24 sites (Figure 4L). Although m^6^A and m^5^C showed more substantial variations, A-to-I alterations were relatively limited in number but still demonstrated consistent changes. These findings establish a foundation for subsequent integrative analysis at the transcript level.

### 3.5 Alternative splicing alterations and integrated analysis with transcript expression and m^6^A modifications in atherosclerosis

Alternative splicing is a key contributor to transcript diversity at the post-transcriptional level. Through selective exon and intron inclusion or exclusion, a single gene can produce multiple mature mRNA isoforms with distinct functional properties, thereby encoding proteins that differ in structure or function (Wright et al., 2022). Based on full-length transcript data derived from DRS, 459 differentially regulated ASEs were identified (Supplementary Figure S5A). Among the alternative splicing categories, SE and RI constituted the largest proportions, followed by AF and alternative 3′splice site/alternative 5′splice site, while MX and alternative last exon events were comparatively less frequent (Supplementary Figure S5A). Across all samples, comparison of PSI and dPSI between known and novel splicing sites demonstrated that known events were detected more frequently and exhibited higher dPSI values. Although MX events were relatively infrequent, they exhibited a broad range of PSI and dPSI values (Supplementary Figures S5B,C).

Subsequently, transcript-level integration of DETs with transcripts involved in differential ASEs was conducted, resulting in the identification of 57 known and 35 novel transcripts exhibiting both differential expression and splicing alterations (Supplementary Figure S5D). The 35 novel transcripts were mapped to their corresponding genes, and functional enrichment analysis of these genes highlighted metabolism-related GO terms and KEGG pathways, including lipid and fatty acid metabolism, thermogenesis, and the peroxisome proliferator–activated receptor signaling pathway (Supplementary Figure S5E). DETs were further intersected with differentially m^6^A-modified transcripts and alternatively spliced transcripts, yielding four transcripts that displayed concurrent changes in expression, m^6^A modification, and splicing (Figure 5A). Notably, the isoform ENSMUST00000110191.10 of *Gcnt2* in the AS group exhibited reduced AF events and harbored a differential m^6^A site within the first exon. This isoform was significantly downregulated in AS, whereas another isoform, ENSMUST00000067778.8, demonstrated relatively increased expression (Zhou et al., 2024). This pattern was accompanied by concurrent changes in m^6^A modification, alternative splicing, and transcript abundance, suggesting an association among these post-transcriptional regulatory features. A comparable phenomenon was observed in *Iigp1* isoforms (Figures 5B,C) (Zeng et al., 2009). Together, these observations indicate that, for a subset of transcripts, differential expression in atherosclerosis is associated with concurrent changes in local RNA modification patterns and alternative splicing.

**FIGURE 5:**
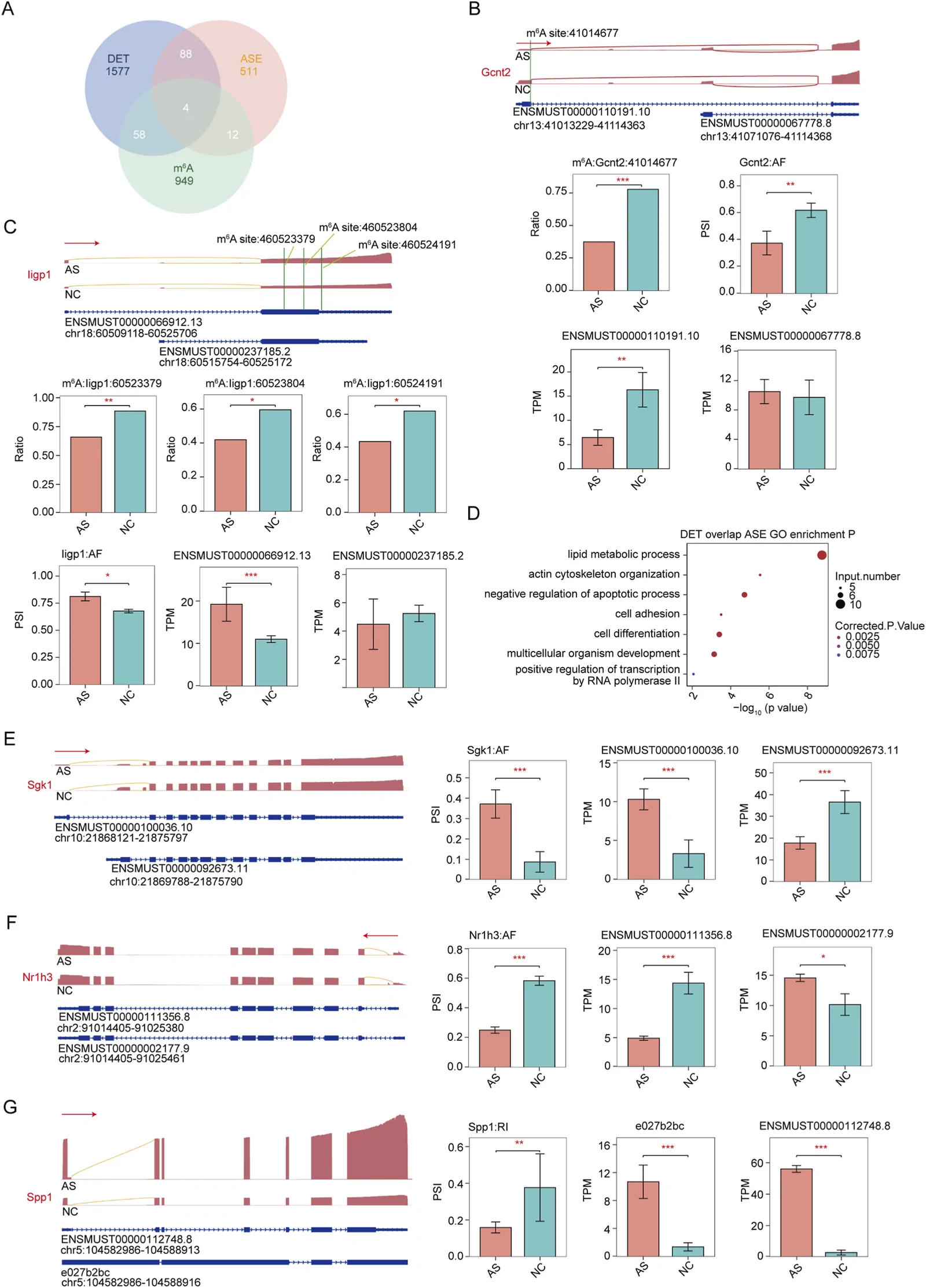
Integrative analysis of isoform abundance, m^6^A modification, and alternative splicing in DRS data. **(A)** Venn diagram illustrating overlap among DETs, alternative splicing events (ASEs), and m^6^A-associated transcripts between AS and NC groups. **(B,C)** Representative examples of *Gcnt2* **(B)** and *Iigp1* **(C)** alternative first exon (AF) events. Top: IGV sashimi plot illustrating AF events and m^6^A-modified sites across transcripts. Bottom: Bar plots summarizing m^6^A ratio at labeled sites, percent spliced-in (PSI) of AF events, and isoform abundance (TPM) in AS and NC. **(D)** GO biological process enrichment analysis of genes corresponding to transcripts overlapping between DETs and ASE-associated transcripts. **(E,F)** Representative examples of *Sgk1* **(E)** and *Nr1h3* **(F)** AF events. Top: IGV sashimi plot illustrating AF events across transcripts. Bottom: Bar plots summarizing PSI of AF events, and isoform abundance (TPM) in AS and NC. **(G)** Representative example of *Spp1* intron retention (RI) event. Top: IGV sashimi plot illustrating RI events across transcripts. Bottom: Bar plots summarizing PSI of RI events and isoform abundance (TPM) in AS and NC. Transcript identifiers with non-standard alphanumeric codes (e.g., e027b2bc) represent novel transcript models reconstructed by the FLAIR/SQANTI3 pipeline that are not present in the Ensembl reference annotation (GRCm39). Previously annotated transcripts are represented using standard Ensembl transcript identifiers (ENSMUST). Data are shown as mean ± SEM (n = 3 biological replicates per group). ***, *P* < 0.001; **, *P* < 0.01; *, *P* < 0.05.

The 56 transcripts identified at the DET–ASE intersection were mapped to their corresponding genes, and GO enrichment analysis of these genes demonstrated significant enrichment in lipid metabolic processes (Figure 5D). For instance, *Sgk1* exhibited a characteristic splicing alteration associated with isoform abundance. In the AS group, the PSI of AF events was significantly increased, accompanied by upregulation of the AF-linked isoform (ENSMUST00000100036.10) and relative downregulation of another isoform (ENSMUST0000092673.11), indicating alternative exon selection and isoform switching in atherosclerotic aortas (Figure 5E) (Voelkl et al., 2018). A similar pattern was observed at the *Nr1h3* locus, where differential AF events were accompanied by downregulation of ENSMUST00000111356.8 and relative upregulation of ENSMUST00000002177.9 in the AS group (Figure 5F). Given that *Nr1h3* encodes liver X receptor alpha, a critical regulator of lipid homeostasis and inflammation, these exon selection shifts and isoform-level alterations may provide structural insights into the differential regulation of the liver X receptor alpha pathway under atherosclerotic conditions (Lu et al., 2025). On the other hand, the osteopontin-encoding gene *Spp1* displayed more complete splicing and reduced RI in atherosclerotic tissue. However, in parallel with increased expression of isoform e027b2bc, the intron-retained isoform ENSMUST00000112748.8 was also significantly upregulated, suggesting that *Spp1* expression may be influenced by other regulatory mechanisms beyond RI events (Figure 5G) (Huang et al., 2024).

### 3.6 DRS identifies atherosclerosis-associated changes in APA and poly(A) tail length

Beyond mRNA modifications and alternative splicing, 3′-end processing of mRNA is another major layer of post-transcriptional regulation. APA regulates 3′UTR architecture by selective poly(A) site usage, thereby affecting mRNA stability and translation efficiency in concert with poly(A) tail length (Gruber and Zavolan, 2019). Quantification of PAS usage demonstrated that genes harboring ≥4 PASs were significantly fewer in the AS group, suggesting reduced complexity of 3′-end site utilization during atherosclerosis (Supplementary Figure S6A). Between-group comparison identified 1,320 significantly altered PASs (Supplementary Figure S6B), and 613 genes showed significant 3′UTR lengthening or shortening (Supplementary Figure S6C). The poly(A) tail length distribution revealed a higher proportion of transcripts with 50 nt tails in the AS group (Supplementary Figure S6D). At the transcript level, more known transcripts than novel transcripts exhibited significant alterations in poly(A) tail length (Supplementary Figure S6E).

Subsequently, gene-level results from DETs, differential m^6^A-modification, poly (A) tail length, and PAS usage were integrated. This analysis identified *Rhoh*, which showed concurrent changes in expression, poly (A) tail length, and APA (Figure 6A). *Rhoh*, which is involved in immune cell signaling and migration, showed significant expression differences in isoform ENSMUST00000201533, potentially associated with 3′-end shifts (Figure 6B) (Kang et al., 2018). Additionally, the *B4galt5* isoform ENSMUST00000109221 exhibited consistent differences in expression, m^6^A modification, and poly(A) tail length, indicating coordinated changes in transcript expression, modification status, and 3′-end characteristics (Figure 6C) (Li et al., 2018). Within the subset with simultaneous changes in expression, APA, and m^6^A, the macrophage-associated complement gene *C1qc* showed transcript 7c93bf07 with coordinated changes in abundance and m^6^A/APA patterns (Figure 6D), consistent with post-transcriptional remodeling during inflammation (Yong et al., 2025). Functional enrichment analysis of genes at the DET-APA intersection revealed significant enrichment in lipid metabolism processes (Figure 6E). Notably, transcripts derived from metabolic genes like *Fasn* (4347bd21 and e716e491), involved in fatty acid synthesis, and *Scp2* (ENSMUST00000106701.2), related to intracellular cholesterol and lipid transport, exhibited strong correlations between major transcript levels and remodeling of 3′-end APA sites (Figure 6F) (Peng et al., 2026; He et al., 2018). These findings support an association between 3′-end site usage and expression changes of lipid metabolism-related genes during atherosclerosis.

**FIGURE 6:**
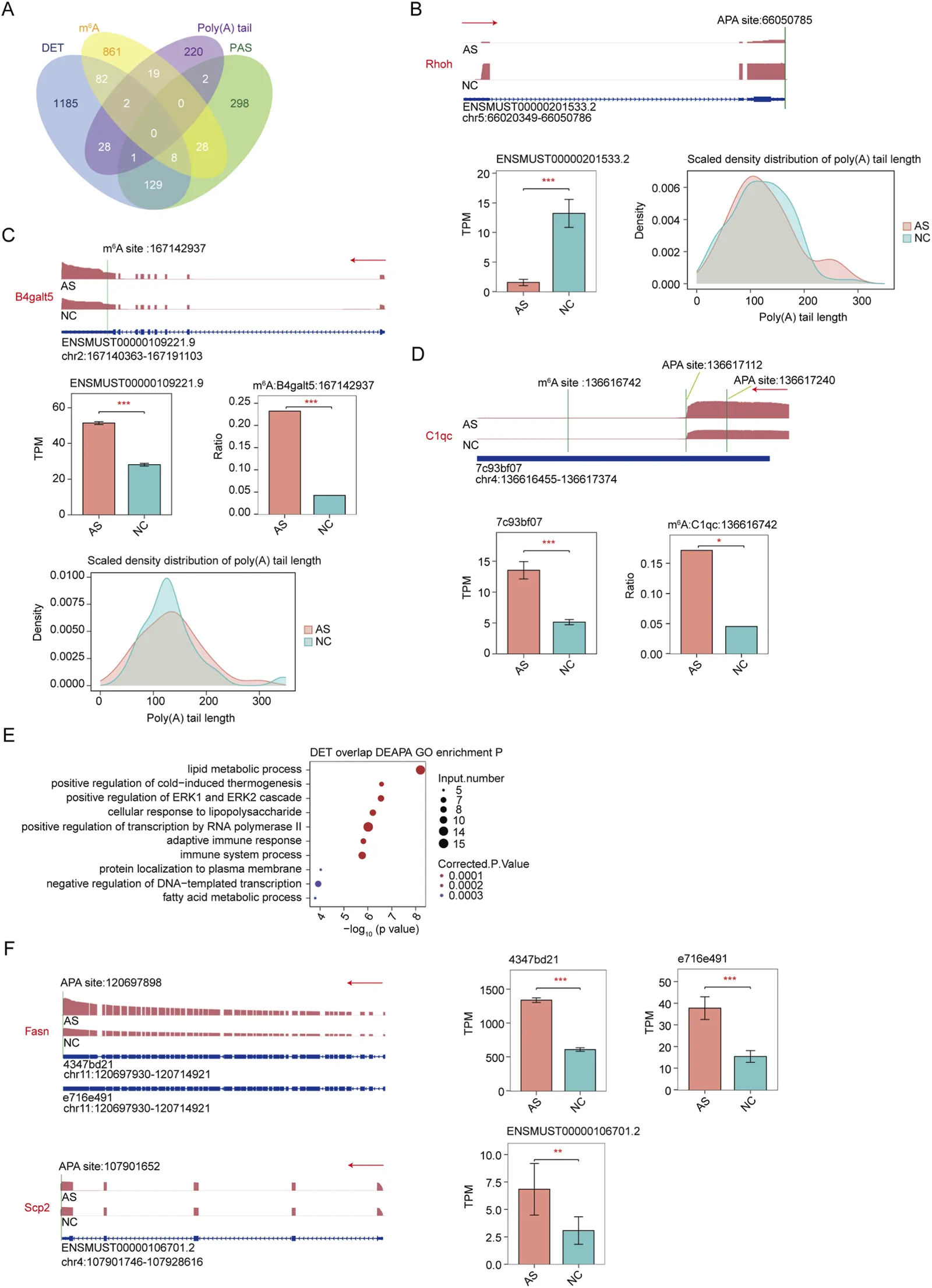
Integrative analysis of isoform abundance, m^6^A modification and 3′-end processing at the gene level. **(A)** Venn diagram illustrating gene-level overlap among genes harboring DETs, differential m^6^A modification, differential poly **(A)** tail length, and differential APA (inferred from polyadenylation site usage) between AS and NC groups. **(B)** Representative example of *Rhoh*. Top: IGV track illustrating transcript structure and APA site; Bottom: Bar plot summarizing transcript abundance (TPM) and density plot illustrating poly **(A)** tail length distributions in AS and NC. **(C)** Representative example of *B4galt5*. Top: IGV track illustrating transcript structure and m^6^A-modified site; Bottom: Bar plot summarizing transcript abundance (TPM) and m^6^A ratio and density plot illustrating poly **(A)** tail length distributions in AS and NC. **(D)** Representative example of *C1qc*. Top: IGV track illustrating transcript structure, APA sites, and m^6^A-modified site; Bottom: Bar plot summarizing transcript abundance (TPM) and m^6^A ratio in AS and NC. **(E)** GO biological process enrichment for genes overlapping DETs and differential APA. **(F)** Representative examples of *Fasn* and *Scp2*. Top: IGV track illustrating transcript structure and APA site; Bottom: Bar plot summarizing transcript abundance (TPM) in AS and NC. Transcript identifiers with non-standard alphanumeric codes (e.g., 7c93bf07) represent novel transcript models reconstructed by the FLAIR/SQANTI3 pipeline that are not present in the Ensembl reference annotation (GRCm39). Previously annotated transcripts are represented using standard Ensembl transcript identifiers (ENSMUST). Data are shown as mean ± SEM (n = 3 biological replicates per group). ***, *P* < 0.001; **, *P* < 0.01; *, *P* < 0.05.

## 4 Discussion

This study employed a diet-induced atherosclerosis model in *Apoe* ^*−/−*^ mice and integrated DRS, ATAC-seq, and bulk RNA-seq to characterize the RNA transcriptional regulatory landscape in the aorta. A first comprehensive analysis of transcript isoform dynamics in this model was conducted using DRS, with multi-layer datasets integrated to delineate RNA reprogramming features, including isoform expression, modification states, and 3′-end regulation (APA and poly (A) tail length). These analyses provide isoform-resolved insights into RNA regulatory mechanisms underlying atherosclerosis.

At the technical level, bulk RNA-seq and ATAC-seq offer valuable information on gene expression and chromatin accessibility, enabling investigation of key pathways and regulatory mechanisms (Wang et al., 2021). However, bulk RNA-seq primarily summarizes transcriptional output at the gene level and often misses isoform-specific variation. Therefore, it cannot fully reflect the complexity of RNA regulation or its functional consequences (Hu et al., 2021). In fact, isoform-level alterations directly contribute to the regulation of essential biological processes, including inflammation, lipid metabolism, and vascular cell function, thereby influencing atherosclerotic plaque formation, progression, and instability and ultimately increasing cardiovascular disease risk (Sachse et al., 2023).

Compared with traditional sequencing approaches, DRS does not depend on reference annotations and enables direct detection of complete transcript structures. The full-length aortic transcriptome map generated by DRS expands existing annotations, identifying 35,587 transcripts, of which 44.1% are novel. Among these, 18,081 transcripts were not covered by reference annotations. Both annotated and novel transcripts exhibited pronounced read enrichment near TSS, indicating consistent transcription initiation features rather than random splicing events or technical noise. The average transcript length was greater in NC than in AS, suggesting transcriptional changes during atherosclerosis. This observation may reflect differences in alternative splicing, 3′-end processing, or shifts in cellular composition.

Furthermore, DRS directly sequences full-length transcripts, facilitating the identification of structural changes and splicing isoforms that may be missed by traditional methods. Notably, many disease-associated DETs did not show changes at the gene level, indicating regulation occurring below the gene level with potential isoform-specific functional implications. Transcript-level analysis reveals isoform-specific regulatory changes that are difficult to detect through gene-level aggregation. Known DETs were primarily associated with immune and inflammatory processes, whereas genes corresponding to novel DETs were more related to lipid metabolism and other energy metabolism-related pathways. These findings support that atherosclerosis involves not only inflammation but also metabolic transcriptional changes that are more evident at the novel transcript level. Integrated ATAC-seq and DRS analyses further demonstrated that expression changes in certain DETs were associated with local chromatin accessibility differences, predominantly at promoter regions. Genes like *Acta2*, *Tagln*, and *Lmod1*, which serve as markers of contractile vascular smooth muscle cells and contribute to vascular wall integrity, exhibited reduced transcript levels and decreased accessibility in AS tissue. This pattern matches the transition from contractile to synthetic vascular smooth muscle cells during atherosclerosis (Nanda and Miano, 2012; Grootaert and Bennett, 2021). These coordinated changes may also be interpreted in the context of established transcriptional regulatory networks. SRF, together with its coactivator myocardin, plays a central role in maintaining the vascular smooth muscle cell contractile program and regulating smooth muscle-associated genes, including *Acta2* and *Tagln* (Wang et al., 2003). In parallel, SREBP transcription factors are key regulators of lipid metabolic programs, including fatty acid and cholesterol metabolism (Shimano and Sato, 2017; Ye and DeBose-Boyd, 2018). These established transcriptional programs provide additional biological context for the coordinated changes in chromatin accessibility and transcript abundance identified by ATAC-seq and DRS.

We used DRS to analyze RNA modifications, alternative splicing, and 3′-end processing related structural transcript changes during atherosclerosis. RNA modifications like m^6^A, m^5^C and A-to-I displayed distinct disease-associated shifts, consistent with their regulatory role in inflammation and metabolism. All three modifications exhibited detectable changes in site distribution and modification levels under atherosclerotic conditions. Integration of modification features with transcript structural alterations provided more interpretable evidence for elucidating regulatory mechanisms. For example, in *Gcnt2* and *Iigp1*, one isoform showed coordinated changes in expression, m^6^A modification, and alternative splicing, whereas another major isoform remained relatively stable. This pattern suggests that, under atherosclerotic conditions, gene regulation may preferentially involve isoform processing rather than simple transcriptional activation or repression (Stopa et al., 2025). Regarding alternative splicing, MX-type events were relatively infrequent but exhibited a broad range of PSI and dPSI values PSI and dPSI shifts, indicating heterogeneous splicing changes across these events. The enrichment of lipid metabolism–related pathways among novel transcripts within the DET–ASE overlap further supports that metabolism-associated splicing remodeling may be more readily detectable in previously unannotated transcript structures or splicing events. Similarly, APA and poly(A) tail length exhibited strong transcript specificity, with multiple transcripts showing selective changes in tail length and APA site usage across groups. Even within the same gene, significant poly(A) tail length differences were observed among isoforms, further demonstrating that 3′-end processing is altered in the atherosclerotic tissue environment and may be associated with changes in mRNA stability and translation efficiency. In line with this, transcripts from *Rhoh*, *B4galt5*, *C1qc*, and lipid metabolism-related genes (*Fasn* and *Scp2*) showed coordinated expression changes together with altered 3′-end processing characteristics. These observations support an association between post-transcriptional regulatory remodeling and core pathological processes, including metabolic dysregulation and inflammatory changes, during atherosclerosis progression (Weber and Noels, 2011).

This study aimed to clarify the post-transcriptional regulatory landscape in atherosclerosis, but several limitations exist. First, while the *Apoe* ^*−/−*^ mice represent a well-established model for atherosclerosis research, findings from this model may be relevant to human atherosclerosis but require further validation, as differences in physiology and metabolism between species may influence the translational applicability of certain observations. Second, the sequencing analyses were based on three pooled biological replicates per group, which may limit statistical power for detecting modest biological effects and does not allow assessment of inter-individual variability at the single-animal level. Third, although GENCODE/Ensembl-based annotation and SQANTI3 classification were used to characterize reconstructed transcripts, the designation of “novel transcripts” in the present study was based on the reference annotation used. Some of these transcripts may therefore reflect incomplete annotation, tissue- or disease-specific expression, low-abundance isoforms, or rare splicing events rather than entirely new biological entities. In addition, although Nanopore DRS enables simultaneous characterization of full-length native RNA molecules and putative RNA modification signals, signal-based modification calling remains technically challenging and may be susceptible to technical variability and false-positive assignments, particularly at single-nucleotide resolution and for specific modification types (Liu-Wei et al., 2024). Individual modification sites should therefore be interpreted cautiously and require orthogonal experimental validation. Finally, the expression and post-transcriptional regulatory alterations of representative transcripts identified through the integrative analyses have not yet been independently validated experimentally. Further targeted validation, including isoform-specific RT-PCR/RT-qPCR and assays of RNA modifications or 3′-end processing, will therefore be required to confirm these observations and determine their biological significance. Future studies integrating targeted functional validation with single-cell, spatial, and high-resolution RNA profiling approaches will help refine the cell-type-specific regulatory mechanisms and functional significance of these findings.

## 5 Conclusion

In summary, this study leveraged DRS technology to integrate multi-omics data and establish a detailed landscape encompassing transcript structure, abundance, RNA modifications, and 3′-end regulation. This integrative strategy illustrates the complexity of post-transcriptional RNA regulation in atherosclerosis and offers a deeper understanding of isoform-specific regulatory changes. These findings provide a conceptual foundation for further investigating disease-associated regulatory mechanisms in atherosclerosis and related cardiovascular diseases, as well as for exploring potential RNA-targeted interventions.

## Data Availability

The datasets of bulk RNA-seq (PRJNA1430266) and ATAC-seq (PRJNA1430688) generated during the current study are available in the NCBI BioProject repository/Supplementary Material.
