# Supplementary material for: Direct RNA sequencing-based multi-omics profiling reveals transcriptional and post-transcriptional remodeling in mice with diet-induced atherosclerosis
Source: Front Cell Dev Biol. 2026 Oct 8;14:1953461. doi: 10.3389/fcell.2026.1953461 (PMC13686839; doi:10.3389/fcell.2026.1953461)
Supplement: Supplementary file 1 [file DataSheet1.docx]

Supplementary Material

**Supplementary Figure S1.** **Analysis of expression abundance between DRS and bulk RNA-seq.** The log_10_ (TPM+1) (TPM, transcripts per million) distributions of expression levels derived from bulk RNA-seq, DRS gene-level quantification, and DRS transcript-level quantification.

**Supplementary Figure S2.** **Global analysis of differentially expressed transcripts (DETs) and differentially expressed genes (DEGs). (A)** Number of DETs classified as known versus novel transcripts. **(B)** Enriched Gene Ontology (GO) biological process terms for genes corresponding to known DETs (top terms). **(C)** Enriched GO biological process terms for genes corresponding to novel DETs (top terms). **(D)** Number of DEGs identified by DRS-seq and bulk RNA-seq. **(E)** Venn diagram illustrating the overlap of DEGs identified by DRS and bulk RNA-seq.

**Supplementary Figure S3.**  **Genome-wide identification of differential chromatin accessibility regions (DARs) via ATAC-seq. (A)** Number of upregulated and downregulated DARs (right panel) and the number of associated genes (left panel). **(B)** Genomic distribution of up-DARs and down-DARs across annotation categories.

**Supplementary Figure S4.**  **Regional distribution of RNA modification sites across transcript features.** Fraction of sites located in 5′ untranslated region (UTR), coding sequence (CDS), and 3′ UTR for m^6^A **(A)**, m^5^C **(B)**, and A-to-I **(C)** across AS and NC.

**Supplementary Figure S5.**  **Global landscape of alternative splicing events (ASEs) and overlap between ASE-associated transcripts and DETs. (A)** Number of significantly altered ASEs across different event types (RI, retained intron; SE, skipping exon; A3, alternative 3′ splice site; A5, alternative 5′ splice site; MX, mutually exclusive exon; AF, alternative first exon; AL, alternative last exon). **(B)** Percent spliced-in (PSI) distribution across ASE types, stratified by known and novel transcripts. **(C)** Differential PSI (dPSI) distribution across ASE types, stratified by known and novel transcripts. **(D)** Venn diagram illustrating overlapping transcripts between DETs and ASE-associated transcripts in known transcript sets (top) and novel transcript sets (bottom), respectively. **(E)** GO biological process (top) and Kyoto Encyclopedia of Genes and Genomes (KEGG) (bottom) enrichment analysis of genes corresponding to novel transcripts overlapping between DETs and ASE-associated transcripts. **, *P* < 0.01; *, *P* < 0.05.

**Supplementary Figure S6.**  **Global analysis of polyadenylation sites (PAS) usage and poly(A) tail length changes in DRS data. (A)** Distribution of PAS number per gene in NC and AS groups. **(B)** Number of differential PAS (Up and Down) between AS and NC groups. **(C)** Number of genes with 3′ UTR length changes (Longer and Shorter) based on differential PAS usage between AS and NC groups. **(D)** Distribution of poly(A) tail lengths in AS and NC groups. **(E)** Number of transcripts with differential poly(A) tail length, stratified by known and novel transcripts.
